# Premarket Pivotal Trial End Points and Postmarketing Requirements for FDA Breakthrough Therapies

**DOI:** 10.1001/jamanetworkopen.2024.30486

**Published:** 2024-08-27

**Authors:** Maryam Mooghali, Joshua D. Wallach, Joseph S. Ross, Reshma Ramachandran

**Affiliations:** 1 Section of General Internal Medicine, Department of Internal Medicine, Yale School of Medicine, New Haven, Connecticut; 2 Yale Collaboration for Regulatory Rigor, Integrity and Transparency, Yale School of Medicine, New Haven, Connecticut; 3 Department of Epidemiology, Rollins School of Public Health, Emory University, Atlanta, Georgia; 4 Department of Health Policy and Management, Yale School of Public Health, New Haven, Connecticut; 5 Center for Outcomes Research and Evaluation, Yale-New Haven Health System, New Haven, Connecticut

## Abstract

**Importance:**

The US Food and Drug Administration (FDA) awards the breakthrough therapy designation to expedite development and review of therapeutics intended to treat serious conditions when preliminary clinical evidence demonstrates potential substantial improvement over existing therapies on clinically significant end points. Under the 21st Century Cures Act of 2016, the FDA is required to publish and routinely update a list of surrogate markers to provide industry sponsors with indication-specific information about end points that were or may be considered for approval. Therapeutics that are granted breakthrough therapy designation can receive accelerated or traditional approval; however, little is known about those approved through the latter pathway, where postmarketing confirmatory studies are typically not required, regardless of the end point used.

**Objective:**

To evaluate the primary end points used in premarket pivotal trials supporting FDA breakthrough therapy–designated approvals and to determine whether postmarketing studies confirming efficacy were required for approvals based on pivotal trials using surrogate markers as primary end points.

**Design, Setting, and Participants:**

This cross-sectional study used data from the Drugs@FDA database for all original breakthrough therapy–designated approvals from inception to December 31, 2023, in the US. The first designation was approved on November 1, 2013. Data analysis was performed in January 2024.

**Main Outcomes and Measures:**

Descriptive analyses were used to characterize the breakthrough therapy–designated indication approval pathways, the primary end points of pivotal efficacy trials, and their postmarketing requirements or commitments.

**Results:**

From 2013 to 2023, the FDA approved 157 original indications with breakthrough therapy designation. Of these, 52 (33%) were granted accelerated approval and 105 (67%) were granted traditional approval. All accelerated approvals were based on pivotal trials using surrogate markers as primary end points and had FDA-required postmarketing studies to confirm efficacy. Of these 52 indications, 51 (98%) were approved based on surrogate end points listed in the FDA table of surrogate end points for the same indication. Among traditional approvals, 61 (58%) were based on pivotal trials using surrogate markers as primary end points, of which 4 (7%) had FDA-required postmarketing studies to confirm efficacy and 39 (64%) were approved based on surrogate end points listed in the FDA table for the same indication.

**Conclusions and Relevance:**

In this cross-sectional study of original FDA breakthrough therapy–designated approvals from 2013 to 2023, trials supporting these approvals often used surrogate markers as primary end points (even when not approved via accelerated approval) and lacked FDA-required postmarketing studies to verify clinical benefit. These findings suggest that requiring postmarketing studies for breakthrough therapy–designated indications approved based on surrogate markers, regardless of approval pathway, may increase patient and clinician certainty of the expected clinical benefit.

## Key Points

**Question:**

What are the premarket end points and postmarketing requirements for US Food and Drug Administration (FDA) breakthrough therapy–designated approvals?

**Findings:**

In this cross-sectional study of 157 original indications with breakthrough therapy designation, all accelerated approvals and 58% of traditional approvals were based on pivotal trials using surrogate markers as primary end points. Whereas all breakthrough therapy–designated accelerated approvals had FDA-required postmarketing studies, only 7% of traditional approvals based on surrogate marker primary end points had such studies.

**Meaning:**

These findings suggest that frequent use of surrogate markers as primary end points in pivotal studies without requiring postmarketing studies to verify clinical benefit could create uncertainties for patients and clinicians around the use of breakthrough therapy–designated drugs.

## Teaser

This cross-sectional study evaluates premarket end points in pivotal clinical trials supporting FDA breakthrough therapy–designated approvals for original indications and postmarketing requirements for approvals based on confirmatory trials using surrogate markers as primary end points.

## Introduction

The US Food and Drug Administration (FDA) determines drug efficacy on the basis of pivotal clinical trials and has increasingly granted approval based on studies using surrogate markers as primary efficacy end points.^1,2,3^ In contrast with clinical outcomes, which measure therapeutic effects by assessing how patients feel, function, or survive, surrogate markers (eg, laboratory measurements or imaging findings) serve as a proxy measure in lieu of directly measuring clinical outcomes and are thought to be predictive of clinical benefits.^4,5^

Under the regulatory pathway of accelerated approval, the FDA explicitly allows the use of surrogate markers “reasonably likely to predict clinical benefit” as primary efficacy end points in clinical trials supporting approval for drugs and biologics addressing serious diseases.^6^ Sponsors of therapeutics for indications that receive accelerated approval are required to conduct postmarketing trials to confirm the expected clinical benefit.^6^ However, surrogate markers may also be used as primary end points in pivotal trials for therapeutics approved outside of accelerated approval, including traditional approval in which the surrogate markers are known to predict clinical benefit.^7^ Nevertheless, prior studies have raised questions around the association between surrogate markers and their predicted clinical outcomes, for both oncology and nononcology therapeutics, finding that surrogate markers used to inform FDA approval for these indications are not consistently correlated with clinical outcomes such as overall survival or quality of life.^8,9,10,11^

Surrogate markers can also be used in the FDA’s determination of another special regulatory pathway, the breakthrough therapy designation. Established under the FDA Safety and Innovation Act of 2012 to expedite the development and review of therapeutics intended to treat serious conditions, the FDA grants the breakthrough therapy designation during the drug development process when preliminary clinical evidence demonstrates potential for substantial improvement over existing therapies on clinically significant end points, including certain surrogate markers.^12,13^ Receipt of this designation grants sponsors eligibility for more frequent meetings and written feedback around the drug’s development, agency commitment “involving senior managers,” and rolling review of application materials as the drug is being developed.^14^ Receipt of the breakthrough therapy designation has also been shown to significantly shorten clinical development periods to a median of 5.2 years compared with 7.2 years for therapeutic approvals using no expedited program.^15^

In prior studies evaluating the breakthrough therapy–designated indications approved in the first years of the program, investigators found that pivotal trials for these indications often were nonrandomized, used surrogate markers as primary end points, and enrolled small numbers of participants.^16,17^ However, little is known about whether the FDA requires additional postmarketing studies to confirm clinical benefit for breakthrough therapy–designated indications approved on the basis of surrogate markers.

Accordingly, we evaluated the primary end points used in pivotal clinical trials supporting all FDA breakthrough therapy–designated approvals for original indications since the inception of the designation in 2012 until December 31, 2023, along with their approval pathways. We also further examined whether the FDA required or requested additional postmarketing studies to confirm clinical efficacy for indications approved based on pivotal trials that used surrogate markers as primary end points. These findings will be particularly informative in the context of recently enacted legislation through the Food and Drug Omnibus Reform Act of 2022, which strengthened the FDA authority over postmarketing requirements for drugs that receive accelerated approval, in part because of prior compliance failures,^18^ but does not address breakthrough therapy–designated indications that receive traditional approval.

## Methods

In accordance with the Common Rule, the Yale School of Medicine deemed this cross-sectional study exempt from ethics review and informed consent because it used public, nonidentifiable data and was not human participant research. The study followed the Strengthening the Reporting of Observational Studies in Epidemiology (STROBE) reporting guideline.

### Data Sources and Study Sample

Using the FDA Breakthrough Therapy Approvals reports,^19^ we identified all original approvals for breakthrough therapy–designated drugs and biologics as of December 31, 2023. Next, we used approval letters in the Drugs@FDA database^20^ to identify the approval pathway (ie, accelerated or traditional approval). For each indication, we identified the pivotal efficacy trials supporting the FDA approval and the corresponding primary end points using the clinical review documents within the drug approval packages, also located in the Drugs@FDA database.

### Assessment of Pivotal Trial End Points

Primary end points were classified as either clinical outcomes or surrogate markers according to the FDA definition of clinical trial end points.^4^ Under the 21st Century Cures Act of 2016, the FDA is required to publish and routinely update a table of surrogate end points to provide industry sponsors with indication-specific information about surrogate markers that were or may be considered by the agency for approval.^7^ For each indication approved based on pivotal trials with only surrogate markers as primary end points, we determined whether the FDA table of surrogate end points included the surrogate marker for the same indication (ie, “Patient Population” in the FDA table).^7^ If the end point was not the same or if the indication was not listed in the FDA table, we determined whether that surrogate marker was listed for another indication.

### Assessment of Postmarketing Requirements and Commitments

For indications approved based on trials using surrogate markers as primary end points, we determined whether approval letters outlined any postmarketing requirements or commitments describing studies explicitly focused on confirming efficacy. Postmarketing requirements are studies that the FDA requires sponsors to complete by a prespecified deadline, whereas postmarketing commitments are studies that sponsors have voluntarily agreed to conduct but are not required by statute or regulation.^21^ We excluded postmarketing requirements describing studies evaluating efficacy in a subpopulation of the patient population (eg, those with a specific demographic background or with a certain genotype) and those focused on pharmacokinetics, pharmacodynamics, safety, or tolerability. For approvals with postmarketing requirements or commitments to confirm efficacy, we then searched the FDA Postmarketing Requirements and Commitments Downloadable Database File^22^ and subsequent approval letters to determine the status of the studies required in the postmarketing setting, using previously described methods.^23^ According to the FDA definition, the status of postmarketing requirements was classified as follows: (1) released (ie, no longer obligated to conduct the study because the study is no longer feasible or would not provide useful data), (2) fulfilled (ie, after reviewing the final study report, the FDA confirmed the fulfillment of the requirements) or submitted (ie, the final study report was submitted to the FDA), (3) terminated (ie, the study was ended before completion, and the final study report has not yet been submitted to the FDA), (4) delayed (ie, study progression was behind the original schedule), (5) ongoing (ie, study progression was according to or ahead of the original schedule), and (6) pending (not initiated).^24^ We then calculated the duration from the approval date until the fulfillment of postmarketing requirements or commitments, if applicable.

### Statistical Analysis

We used descriptive analyses to characterize the breakthrough therapy–designated indications included in our sample, the primary end points of pivotal efficacy trials, and their postmarketing requirements or commitments. Data analysis was performed in January 2024 using Excel, version 16.79 (Microsoft Inc).

## Results

### Characteristics of Breakthrough Therapy–Designated Indications

Since the inception of the breakthrough therapy designation in 2012 until December 31, 2023, the FDA approved 157 original indications with this designation, with the first indication being approved on November 1, 2013. Of these indications, 52 (33%) were granted accelerated approval and 105 (67%) were granted traditional approval (Table 1). Among the 52 indications granted accelerated approval, 29 (56%) were drugs and 23 (44%) were biologics; 49 (94%) were approved for hematologic and oncologic indications, 1 (2%) for an infectious disease, 1 (2%) for a neurologic and psychiatric indication, and 1 (2%) for a genetic and metabolic disease. Among those granted traditional approval, 72 (69%) were drugs and 33 (31%) were biologics; 38 (36%) were approved for hematologic and oncologic indications, 19 (18%) for infectious diseases, 14 (13%) for neurologic and psychiatric indications, 7 (7%) for pulmonologic indications, and 13 (12%) for other indications.

**Table 1.** Characteristics and Trial End Points for FDA-Approved Breakthrough Therapy–Designated Indications, 2013 to 2023

Characteristic	No. (%) of FDA approvals (N = 157)	
	Accelerated approval (n = 52)	Traditional approval (n = 105)
Approval year		
2013	1 (2)	2 (2)
2014	4 (8)	5 (5)
2015	5 (10)	7 (7)
2016	4 (8)	5 (5)
2017	4 (8)	16 (15)
2018	4 (8)	14 (13)
2019	6 (12)	8 (8)
2020	9 (17)	16 (15)
2021	7 (13)	12 (11)
2022	5 (10)	11 (10)
2023	3 (6)	9 (9)
Class		
Drug	29 (56)	72 (69)
Biologic	23 (44)	33 (31)
Therapeutic area		
Autoimmune, musculoskeletal, and dermatology	0	5 (5)
Cardiovascular, diabetes, and hyperlipidemia	0	4 (4)
Hematology and oncology	49 (94)	38 (36)
Infectious disease	1 (2)	19 (18)
Neurology and psychiatry	1 (2)	14 (13)
Pulmonology	0	7 (7)
Other^a^	1 (2)	13 (12)
Primary end point of the trial supporting approval		
≥1 clinical end points	0	44 (42)
Only surrogate marker	52 (100)	61 (58)

Abbreviation: FDA, US Food and Drug Administration.

^a^ Included genetic and metabolic diseases, gastroenterology, nephrology, ophthalmology, and pain medicine.

### Breakthrough Therapy–Designated Indications With Accelerated Approval

All 52 breakthrough therapy–designated indications that received accelerated approval had pivotal trials that used surrogate markers as primary end points. The surrogate markers used as primary efficacy end points in pivotal trials supporting the 51 accelerated approval indications (98%) were listed in the FDA table of surrogate end points for the same indication (Table 2). One indication (2%) (to treat PIK3CA-related overgrowth spectrum) was not listed in the FDA table, and the corresponding surrogate marker used in the pivotal trial (reduction in the sum of measurable target lesion volume) was not listed in the FDA table for any other indication (Table 3).

**Table 2.** Consistency of Surrogate End Points Used in Trials Supporting Breakthrough Therapy–Designated Approvals With the FDA Table for Surrogate End Points, 2013 to 2023

Inclusion of indication in the FDA surrogate end point table	No. (%) of FDA approvals	
	Accelerated approval (n = 52)	Traditional approval (n = 61)
Yes		
Surrogate end point listed for the same indication	51 (98)	39 (64)
Surrogate end point listed for a different indication	0	2 (3)
Surrogate end point not listed	0	1 (2)
No		
Surrogate end point listed for a different indication	0	10 (16)
Surrogate end point not listed	1 (2)	9 (15)

Abbreviation: FDA, US Food and Drug Administration.

**Table 3.** Breakthrough Therapy–Designated Indications Approved Based on Trials Using Surrogate End Points That Were Not Consistent With End Points Listed by the FDA

Drug	Indication	Surrogate end point used in the trials supporting approval		Surrogate end points mentioned in FDA table for the specific indication
		End point details	End point listed in FDA table^a^ for a different indication?	
Ayvakit	Treatment of adults with unresectable or metastatic gastrointestinal stromal tumor	ORR	Yes	PFS, DFS
Jelmyto	Treatment of adult patients with low-grade upper tract urothelial cancer	Complete response, based on ureteroscopic and local pathology assessment, and durability of complete response	Yes	PFS, ORR
Rukobia	In combination with other antiretroviral(s) for the treatment of HIV-1 infection in heavily treatment-experienced adults with multidrug-resistant HIV-1 infection after failure of the current antiretroviral regimen due to resistance, intolerance, or safety considerations	Adjusted mean decline in HIV-1 RNA (mean change in log_10_ RNA)	No	Undetectable plasma HIV RNA; serum HIV antibody; >0.5-log reduction in plasma HIV RNA
Gamifant	Treatment of primary hemophagocytic lymphohistiocytosis	ORR	Yes	Indication not listed in the FDA table
Elzonris	Treatment of blastic plasmacytoid dendritic cell neoplasm	Complete response or clinical complete response rate with minimal residual skin abnormality; rate based on tumor response criteria for bone marrow, peripheral blood, skin, nodal masses, spleen, and liver	Yes	Indication not listed in the FDA table
Turalio	Treatment of adult patients with symptomatic tenosynovial giant cell tumor	ORR	Yes	Indication not listed in the FDA table
Koselugo	Treatment of neurofibromatosis type 1	ORR	Yes	Indication not listed in the FDA table
Rezurock	Treatment of chronic graft-vs-host disease after failure of at least 2 prior lines of systemic therapy	ORR	Yes	Indication not listed in the FDA table
Nexviazyme	Treatment of late-onset Pompe disease	Change in percent predicted FVC in upright position	Yes	Indication not listed in the FDA table
Fyarro	Treatment of adult patients with locally advanced unresectable or metastatic malignant perivascular epithelioid cell tumor	ORR and DOR	Yes	Indication not listed in the FDA table
Tafinlar	In combination with trametinib, for the treatment of pediatric patients aged ≥1 y with low-grade glioma with a BRAF V600E mutation who require systemic therapy	ORR	Yes	Indication not listed in the FDA table
Ogsiveo	Treatment of adult patients with progressing desmoid tumors that require systemic treatment	PFS	Yes	Indication not listed in the FDA table
Vijoice	Treatment of adult and pediatric patients aged ≥2 y with severe manifestations of PIK3CA-related overgrowth spectrum who require systemic therapy	Reduction in the sum of measurable target lesion volume	No	Indication not listed in the FDA table
Xuriden	Treatment of hereditary orotic aciduria	Stable hematologic value	No	Indication not listed in the FDA table
Krintafel	For the radical cure (prevention of relapse) of *Plasmodium vivax* malaria	No. of participants with recurrence-free efficacy	No	Indication not listed in the FDA table
Azedra	Treatment of adult and pediatric patients aged ≥12 y with iobenguane scan–positive, unresectable, locally advanced, or metastatic pheochromocytoma or paraganglioma who require systemic anticancer therapy	Proportion of patients who experienced a ≥50% reduction of all antihypertensive medication(s)	No	Indication not listed in the FDA table
Livmarli	Treatment of cholestatic pruritus associated with Alagille syndrome	Change in fasting serum bile acid levels in participants who had a ≥50% reduction in serum bile acid	No	Indication not listed in the FDA table
Livtencity	Treatment of posttransplant CMV infection or disease that does not respond (with or without genetic mutations that cause resistance) to available antiviral treatment for CMV	Percentage of participants who achieved confirmed clearance of plasma CMV DNA (CMV viremia clearance)	No	Indication not listed in the FDA table
Xenpozyme	Treatment of acid sphingomyelinase deficiency	Percentage of predicted DLCO, spleen volume, liver volume, and platelet count	No	Indication not found in the FDA table
Tzield	To delay the onset of stage 3 type 1 diabetes in adults and pediatric patients aged ≥8 y with stage 2 type 1 diabetes	Rate of new diabetes per year	No	Indication not listed in the FDA table
Izervay	Treatment of geographic atrophy secondary to age-related macular degeneration	Change in geographic atrophy as measured by fundus autofluorescence	No	Indication not listed in the FDA table
Pombiliti	In combination with Opfolda, for the treatment of adult patients with late-onset Pompe disease (lysosomal acid α-glucosidase deficiency) weighing ≥40 kg and whose symptoms are not improving with their current enzyme replacement therapy	Change in 6-min walk distance and sitting FVC (percentage of predicted)	No	Indication not found in the FDA table
Opfolda	In combination with Pombiliti, for the treatment of adult patients with late-onset Pompe disease (lysosomal acid α-glucosidase deficiency) weighing ≥40 kg and whose symptoms are not improving with their current enzyme replacement therapy	Change in 6-min walk distance and sitting FVC (percentage of predicted)	No	Indication not listed in the FDA table

Abbreviations: CMV, cytomegalovirus; DFS, disease-free survival; DLCO, diffusing capacity of the lungs for carbon monoxide; FDA, US Food and Drug Administration; FVC, forced vital capacity; ORR, objective response rate; PFS, progression-free survival.

^a^ Refers to the FDA table for surrogate end points.

For all 52 breakthrough therapy–designated indications granted accelerated approval, the FDA had required postmarketing studies to confirm efficacy. As of January 20, 2024, the status of these 52 requirements was as follows: 6 (12%) were released (ie, no longer obligated to conduct the study as the study was no longer feasible or would not provide useful data), 26 (50%) were fulfilled (ie, after reviewing the final study report, the FDA confirmed the fulfillment of the requirements), 1 (2%) was submitted (ie, final study report was submitted to the FDA), 5 (10%) were delayed (ie, study progression was behind the original schedule), 7 (13%) were ongoing (ie, study progression was according to or ahead of the original schedule), and 7 (13%) were pending (not initiated). Among the 26 fulfilled requirements, the median duration from approval to fulfillment was 27.6 (IQR, 17.1-36.4) months. The median duration since approval for the 7 indications with pending (not initiated) postmarketing efficacy requirements was 13.3 (IQR, 9.2-15.3) months, as of January 20, 2024.

### Breakthrough Therapy–Designated Indications With Traditional Approval

**Figure 1.**
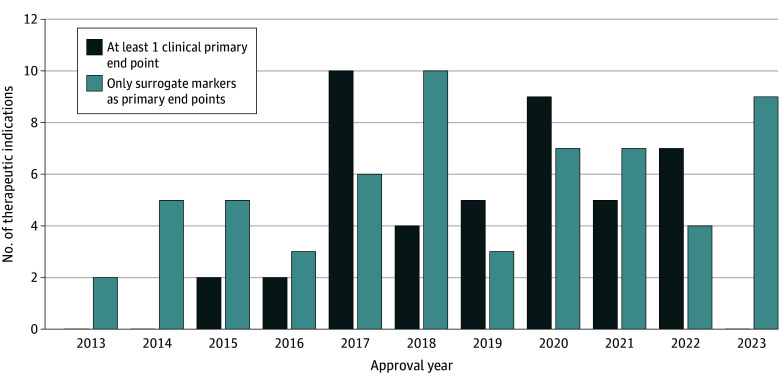
End Points for US Food and Drug Administration Breakthrough Therapy–Designated Indications Granted Traditional Approval, 2013 to 2023 (n = 105)

**Figure 2.**
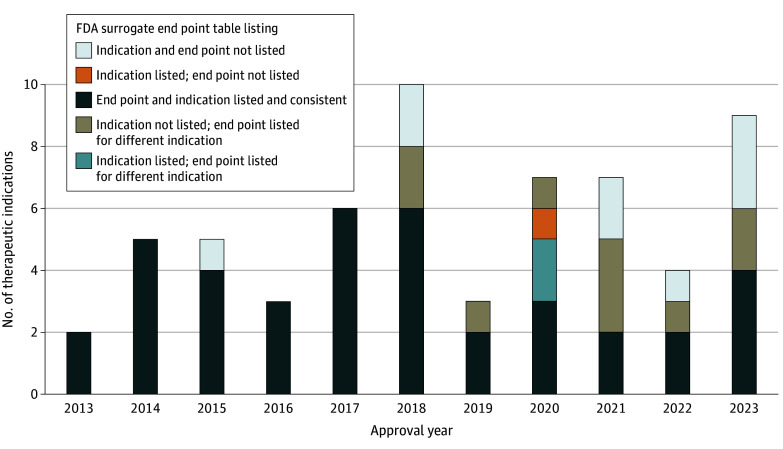
Surrogate End Points for US Food and Drug Administration (FDA) Breakthrough Therapy–Designated Indications Granted Traditional Approval, 2013 to 2023 (n = 61)

Among the 105 breakthrough therapy–designated indications that received traditional approval, 44 (42%) were approved based on pivotal trials that used at least 1 clinical outcome as a primary end point and 61 (58%) were approved based on pivotal trials that used only surrogate markers as primary end points (Figure 1). Among the 61 indications approved based on surrogate markers, 42 (69%) were included in the FDA table of surrogate end points. Of these 42 indications, 39 (93%) were approved based on surrogate end points listed for the same indication, 2 (5%) were approved based on surrogate end points that differed from the end point listed in the FDA table for that specific indication but were listed for another indication, and 1 (2%) was approved based on an end point that was not listed in the FDA table for any indication. Of the 61 indications, 19 (31%) were not listed in the FDA table; of these, 10 (53%) were approved based on surrogate end points listed in the FDA table for a different indication, and 9 (47%) were approved based on surrogate end points that were not included in the FDA table for any indications (Figure 2).

Of the 61 breakthrough therapy–designated indications granted traditional approval based on pivotal trials using surrogate markers as primary end points, 29 (48%) were for hematologic and oncologic indications. The surrogate markers used to support approval of these indications included progression-free survival, overall response rate, complete remission rate, complete response rate, duration of severe neutropenia, major molecular response, event-free survival, and reduction of antihypertensive medications. Among these end points, a single end point corresponding to 1 indication (3%) (reduction of all antihypertensive medication for the treatment of unresectable, locally advanced, or metastatic pheochromocytoma or paraganglioma) was not listed in the FDA table for any indication. A total of 13 of 61 approvals (21%) were for infectious disease indications based on trials that used surrogate markers as primary end points, including sustained virologic response, proportion of patients with a 0.5 or greater log_10_ decrease in viral load, mean adjusted decrease in viral load, viremia clearance, and parasitemia clearance. Of these 13 approvals, 3 end points—corresponding to 3 indications (23%) (parasitemia clearance for radical cure of *Plasmodium vivax* malaria, mean adjusted decrease in viral load for treatment of HIV-1 infection, and viral clearance for treatment of posttransplant cytomegalovirus infection)—were not listed in the FDA table for any indications. Of the 61 approvals, 7 (11%) were for pulmonology indications, based on change in percent predicted forced expiratory volume in 1 second or change in forced vital capacity; both surrogate markers were listed in the FDA table for the same approved indications.

Among the 61 breakthrough therapy–designated indications granted traditional approval based on pivotal trials using surrogate markers as primary end points, 4 (7%) had FDA-required postmarketing studies to confirm efficacy. These requirements included the following: (1) completion and submission of the final report for an ongoing observational study assessing resistance and durability of response to direct-acting antiviral agent therapy for chronic hepatitis C virus infection; (2) conducting a new study evaluating disease progression or survival for chemotherapy-induced myelosuppression; (3) conducting a safety analysis with efficacy results when treating chronic graft-vs-host disease, using data from prior clinical trials; and (4) completion and submission of the final report for a randomized clinical trial and a single-group study to evaluate the efficacy and safety results for the treatment of paroxysmal nocturnal hemoglobinuria. As of January 20, 2024, the status of these 4 requirements was as follows: 1 study (25%) was fulfilled, 1 study (25%) was submitted, 1 study (25%) was pending (not initiated), and 1 study (25%) could not be located. Additionally, 3 indications had postmarketing efficacy requirements only in a subpopulation of the indicated population (eTable in Supplement 1).

## Discussion

In this study of FDA breakthrough therapy–designated approvals between 2013 and 2023, two-thirds were based on pivotal trials that used surrogate markers as primary efficacy end points. For breakthrough therapy–designated indications granted traditional approval, more than half were based on surrogate markers, many of which are not currently listed in the FDA table of surrogate end points meant to guide sponsors about which surrogate markers have been or may be considered for indication approvals. Moreover, unlike with accelerated approvals in which the FDA required postmarketing studies to confirm the expected clinical benefit for all breakthrough therapy–designated indications, traditional approvals based on surrogate markers were generally not accompanied by FDA-required postmarketing efficacy studies. These findings underline the uncertainty that patients and clinicians may face when assessing the clinical benefits of breakthrough therapy–designated indications without the opportunity for further evidence to be generated after approval to confirm such clinical benefits.

Our study suggests that over a decade after the inception of the breakthrough therapy designation, the majority of the designated indications were approved based on clinical trials using only surrogate markers as the primary efficacy end points, including approvals through both the accelerated and traditional approval pathways. These findings were consistent with previous research examining approvals during the early years of the program.^16^ However, previous studies have raised concerns around the lack of a strong correlation between surrogate markers and clinical outcomes,^8,9,11,25^ and the FDA has acknowledged that surrogate markers may provide incomplete information about the clinical benefits of therapeutics.^26^ Certain treatments that have demonstrated clinical benefit for one indication might be ineffective or even harmful for others, often due to potentially unmeasured effects by the surrogate markers.^26,27,28^

Despite recent research showing greater overall survival for breakthrough therapy–designated vs nondesignated cancer therapeutics,^29^ concerns have also been raised that the designation may imply an exceptional magnitude of clinical benefit, creating a perception among the public as well as clinicians that these medications substantially improve patient outcomes by providing a cure for serious illnesses or improving patients’ quality of life or survival.^30,31^ For instance, evidence suggests that a substantial proportion of physicians misinterpret the meaning of the breakthrough therapy designation in terms of the evidentiary standards, including that the designation automatically confers eligibility for receiving accelerated approval.^32^ Thus, when breakthrough therapy–designated indications are approved based on surrogate end points instead of clinical outcomes, without consistent postmarketing requirements to confirm benefit, this may hinder accurate and informed decision-making by patients and clinicians.

In fact, we found that the FDA consistently required additional studies to confirm clinical benefit for the originally indicated population only when breakthrough therapy–designated drugs were granted accelerated approval on the basis of pivotal trials using surrogate markers. For most breakthrough therapy–designated drugs using surrogate markers approved under the traditional pathway, the FDA did not require such postmarketing efficacy studies. Therefore, the FDA should encourage sponsors to use clinical outcomes as primary end points of pivotal trials, especially if the indication is being considered for traditional approval. Furthermore, for these approved indications awarded the breakthrough therapy designation with pivotal trials using surrogate markers as primary end points, the FDA should require sponsors to confirm the clinical benefits in the postmarket setting, regardless of approval pathway.^33^ This includes the traditional approval pathway where if surrogate end points are used as the basis of approval, they are typically considered to be validated.^4^ However, prior studies on both oncology and nononcology indications have demonstrated that surrogate end points used as the basis of traditional approval often lack evidence or strong evidence of association with clinical outcomes,^9,11^ underscoring the need for postmarketing efficacy studies to confirm clinical benefits for traditional approvals based on pivotal trials using surrogate markers. To further improve transparency around these approvals, the FDA could include further justification for the approval decisions, particularly when they do not require postmarketing efficacy studies. Finally, for those breakthrough therapy–designated treatments approved by the FDA based on surrogate markers as primary end points, the agency could require the surrogate marker end points to be among those listed for the specific indication within the FDA table of surrogate end points.

In addition to requiring postmarketing studies to confirm clinical benefit for breakthrough therapies approved on the basis of studies using surrogate markers as primary end points, regardless of approval pathway, the FDA should also enable their timely completion. The Food and Drug Omnibus Reform Act of 2022 has awarded the FDA several authorities to do so with accelerated approval while also requiring additional transparency from manufacturers regarding the status of confirmatory studies.^18^ Similar policies could also be established to address parallel concerns around residual uncertainty of clinical benefit of breakthrough therapy–designated indications approved based on surrogate markers as primary end points.

### Limitations

This study has several limitations. First, we only examined original indication approvals, whereas the breakthrough therapy designation could also be used for supplemental indications. Second, we relied on publicly available data, including the FDA table of surrogate end points, and did not have access to communications between the sponsors and the agency that might not be included as part of the approval packages and could have informed the selection of primary end points. Finally, we only evaluated the pivotal efficacy trials, which are the primary evidence informing FDA approval; however, the FDA may consider other studies and nonpublicly available data from the sponsors as supplementary efficacy data for its regulatory decision-making.

## Conclusions

This cross-sectional study of FDA breakthrough therapy–designated indication approvals between 2013 and 2023 showed that pivotal trials supporting the agency’s approvals for these indications often used surrogate markers as primary end points, even when not approved via the accelerated approval pathway, and lacked FDA-required postmarketing studies to confirm the expected clinical benefit. These concerns highlight the uncertainties for patients and clinicians around the quality of evidence supporting FDA approvals of breakthrough therapy–designated indications. Requiring postmarketing requirements for breakthrough therapy–designated indications approved based on surrogate markers, regardless of approval pathway, would ensure that clinical benefits are confirmed.

## Notes

Supplement 1. **eTable.** Excluded Indications With Postmarketing Efficacy Requirements in a Subpopulation of the Indicated Population

Supplement 2.
Data Sharing Statement
