# Supplementary material for: Premarket Pivotal Trial End Points and Postmarketing Requirements for FDA Breakthrough Therapies
Source: JAMA Netw Open. 2024 Aug 27;7(8):e2430486. doi: 10.1001/jamanetworkopen.2024.30486 (PMC11350476; doi:10.1001/jamanetworkopen.2024.30486)
Supplement: Supplement 1. — eTable. Excluded Indications With Postmarketing Efficacy Requirements in a Subpopulation of the Indicated Population [file jamanetwopen-e2430486-s001.pdf]

Supplementary Online Content

Mooghali M, Wallach JD, Ross JS, Ramachandran R. Premarket pivotal trial end points and postmarketing requirements for FDA breakthrough therapies. *JAMA Netw Open*. 2024;7(8):e2430486. doi:10.1001/jamanetworkopen.2024.30486

eTable. Excluded Indications With Postmarketing Efficacy Requirements in a Subpopulation of the Indicated Population

This supplementary material has been provided by the authors to give readers additional information about their work.

© 2024 Mooghali M et al. *JAMA Network Open.*

eTable. Excluded Indications With Postmarketing Efficacy Requirements in a Subpopulation of the Indicated Population

Drug Name	Approval date	Approval Pathway	Indication	Efficacy Postmarketing Requirements
Sovaldi	12/6/2013	Traditional approval	Treatment of chronic hepatitis C infection	Submit the final report and datasets including next generation sequencing for the ongoing trial P7977-2025 in order to identify treatment-emergent substitutions and to obtain additional safety and efficacy data in this population with hepatocellular carcinoma meeting Milan criteria awaiting liver transplantation.
Epclusa	6/28/2016	Traditional approval	Treatment of adult patients with chronic hepatitis C virus genotypes 1, 2, 3, 4, 5 or 6 infection:<br>- without cirrhosis or with compensated cirrhosis<br>- with decompensated cirrhosis for use in combination with ribavirin	Conduct a trial in hepatitis C virus genotype 3 infected subjects with cirrhosis treated with sofosbuvir and velpatasvir to determine if the addition of ribavirin improves the efficacy (i.e., sustained virologic response rate) and reduces the rate of virologic failure.
Besponsa	8/17/2017	Traditional approval	Treatment of adults with relapsed or refractory B-cell precursor acute lymphoblastic leukemia (ALL)	Conduct a randomized trial of at least 2 dose levels of inotuzumab ozogamicin in patients with relapsed or refractory acute lymphoblastic leukemia who are potential candidates for hematopoietic stem cell transplantation (HSCT) and at high risk for developing veno-occlusive disease (VOD). High risk is defined as patients with prior HSCT, ongoing or prior liver disease, older patients (≥ 55 years), or later salvage line (Salvage ≥ 2). Safety parameters will include hepatic VOD, transplant related mortality (non-relapse mortality), and non-transplant related mortality. Descriptive analyses of safety and efficacy (including achievement of minimal residual disease [MRD]-negativity) will be conducted for the intent-to-treat population and the per-protocol population that excludes patients who do not proceed to HSCT. The study will include sufficient clinical pharmacokinetic sampling to analyze the exposure-response relationship for efficacy and safety. Submit the complete clinical study report and datasets.

© 2024 Mooghali M et al. *JAMA Network Open.*
