# Supplementary material for: Premarket Pivotal Trial End Points and Postmarketing Requirements for FDA Breakthrough Therapies
Source: JAMA Netw Open. 2024 Aug 27;7(8):e2430486. doi: 10.1001/jamanetworkopen.2024.30486 (PMC11350476; doi:10.1001/jamanetworkopen.2024.30486)
Supplement: Supplement 2. — Data Sharing Statement [file jamanetwopen-e2430486-s002.pdf]

Data Sharing Statement

Mooghali. Premarket Pivotal Trial End Points and Postmarketing Requirements for FDA Breakthrough Therapies. *JAMA Netw Open*. Published August 27, 2024. doi:10.1001/jamanetworkopen.2024.30486

Data
Data available: No

Additional Information
Explanation for why data not available: Relevant data are available on reasonable request from the corresponding author.
